# Various Forms of Cardiac Rehabilitation and Their Effect on Frailty Syndrome in Cardiac Patients—A Systematic Review

**DOI:** 10.3390/healthcare12232401

**Published:** 2024-11-29

**Authors:** Magdalena Wójciak, Natalia Świątoniowska-Lonc, Kinga Węgrzynowska-Teodorczyk

**Affiliations:** 1 Centre for Heart Diseases, 4th Military Hospital, 50-981 Wroclaw, Poland; natalia.swiat@o2.pl (N.Ś.-L.); kinga.wegrzynowska@awf.wroc.pl (K.W.-T.); 2 Faculty of Physiotherapy, Wroclaw University of Health and Sport Sciences, 51-612 Wroclaw, Poland

**Keywords:** cardiac rehabilitation, frailty syndrome, the elderly, movement-based rehabilitation, training with frailty

## Abstract

**Background:** The growing population of the elderly is accompanied by an increase in the number of people with frailty syndrome. Apart from advanced age, the occurrence of cardiovascular diseases is also one of the agents favorable to frailty that is a factor aggravating the disease prognosis. However, it is worth noting that this is an agent changeable by, i.a., movement rehabilitation. Cardiac rehabilitation (CR) based on comprehensiveness, early implementation, and multiplicity is standard intervention in patients with cardiovascular diseases. In cardiac patients with accompanying frailty or pre-frailty syndrome, it is worth making efforts to upgrade the CR program. **Methods:** Studies on the effect of cardiac rehabilitation or its modifications on the level of frailty of patients with cardiovascular disease were analyzed. **Results:** Training programs based on endurance training should be supplemented by resistance, balance, mobility, and respiratory exercises. Furthermore, it is important to educate patients about the need to increase daily physical activity. This review presents different approaches to CR (traditional CR, multicomponent training, training with a HAL (Hybrid Assistive Limb), and prehabilitation) and their impact on frailty score. **Summary:** Early implemented and comprehensive rehabilitation allows for the improvement of the clinical state and for a decrease in overall frailty. It also guarantees safety during everyday activities. It is crucial for the fitness of the elderly to encourage them to increase daily physical activity and to exercises at home.

## 1. Introduction

One of the problems of the modern world is the ageing population. According to data from the WHO (World Health Organization), in 2019, there were 1 billion people above 60 years old, and it is estimated that this number will increase to 1.4 billion in 2030 and to 2.1 billion in 2050. This occurrence is not observed only in developing countries [1] but in all European countries, and it will escalate [2].

Advanced age contributes to the occurrence of frailty syndrome. There are circa 10% (4–59% depending on the criteria) of people older than 65 who are affected by this problem. Among people over 80, that is about ¼ of the population [3]. It is worth noting that the presence of frailty syndrome is related to cardiovascular disease incidence and mortality from cardiac causes (coronary disease is 49% more common, and heart failure is 72% more so). Indeed, frailty syndrome can be the result of the others diseases weakening the organism, e.g., heart failure [4]. The occurrence of frailty is one of the agents of an unfavorable prognosis regarding disease progression and mortality [5,6].

Kojima highlights the lack of a consistent definition of frailty syndrome. Those formulated up to this time have common features included in the WHO’s definition [3]. Frailty syndrome can be defined as a clinical state of decline in physiological reserves in ageing people. In 2016, the WHO [7] highlighted the frailty phenotype (FP), created by Fried et al. [8] and the frailty index (FI), by Mitnitski et al. [9] as instruments for frailty assessment [7].

It is necessary to observe three or more phenotypic criteria to identify frailty according to Fried et al. [8], and one or two for pre-frailty syndrome. Those criteria are: weakness of hand grip, decrease of walking speed, low level of physical activity, low energy or self-reported exhaustion, and unintentional weight loss [7,8].

Mitnitski’s approach is based on deficits related to age and on the proportion of occurring (noted as 1) to non-occurring (noted as 0) ones in an elderly person. Frailty syndrome is described as an accumulation of health deficits. The FI helps predict negative health implications and can be used in different ageing studies [3,7,9].

Most frailty criteria relate to the ageing process and to its accompanying decline of physical fitness and muscle strength. Consideration should be given to the fact that these factors can be modified and reduced by employing physical training. Because of that, the aim of the present publication is to assess the impact of cardiac rehabilitation on frailty syndrome based on the available literature. Therefore, this systematic review aimed to answer the question of whether and how cardiac rehabilitation influences the incidence of frailty syndrome among patients with cardiovascular disease.

It is standard procedure for cardiac patients to apply cardiac rehabilitation (CR) based on, i.a., comprehensiveness, early implementation, and multiplicity. The CR program includes physical activity, nutrition, healthy lifestyle education, and secondary prevention of disease. In the course of physical rehabilitation, i.a., endurance training supplemented with mobility training is used. Endurance training aims to improve the cardiovascular system and the muscles’ ability to work for extended periods of time. Mobility training is based on exercises which increase range of motion and stabilization of joints and has aims to improve control of the joints. CR should be started immediately after cessation of absolute contraindications, like life-threatening conditions or destabilization of the clinical state. During the in-hospital stage, physical exercises are applied to counteract the results of inactivity, adapt the cardiovascular system to physical activity, make patients independent, and also to teach them about a healthy lifestyle. The second stage of rehabilitation—the post-hospital stage—can be realized in stationary, outpatient, or home (telerehabilitation) conditions. Cardiopulmonary system improvement, increase of physical effort tolerance, reduction of risk of occurrence, and exacerbation of cardiovascular disease and all-cause mortality as well as improvement of quality of life are the goals of post-hospital CR. The aim of third stage of CR is to maintain the obtained results by the self-directed physical activity of the patient [10,11,12]. Constant or interval endurance aerobic training is recommended in the second and third stages of CR. Each session should include a warm-up, 20–60 min of a main part, and a cool-down. There should be 3–5 sessions a week with an intensity at 40–70% of the heart rate reserve or 50–80% of exercise capacity. If endurance training is well-tolerated by patients, introduce resistance training performed 2–3 times a week with a load of 30–60% of 1 RM (repetition maximum) with exercises in 1–3 sets. Breathing exercises are also an important part of a training session to improve respiratory capacity and oxygenate the body [12,13].

## 2. Material and Methods

**Figure 1:**
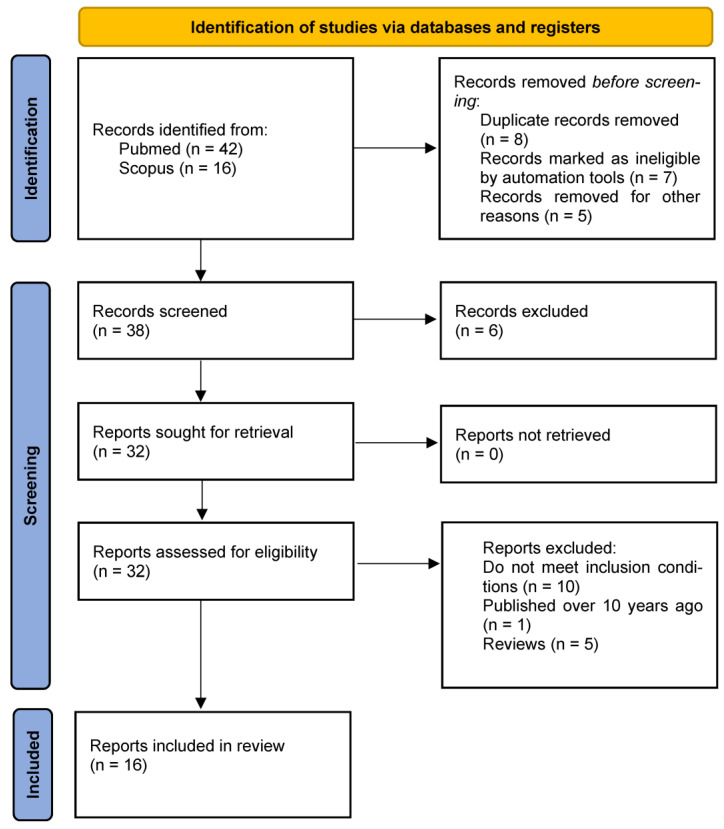
Study flow chart.

Academic publications found on PubMed and Scopus comprise the material used to write this article (Figure 1). During the search for texts, we focused on those studies published within the last 10 years—the analyzed research was published between 2014 and 2024.

From 32 articles, 16 publications presenting the results of research into the impact of CR and its repercussions on frailty syndrome are included in the analysis (Table 1). A total of 16 articles are rejected: 10—do not meet the inclusion conditions, 1—is published over 10 years ago, and 5—are reviews. The words, phrases, and their combinations that were searched for: “frailty”, “frailty syndrome”, “cardiac rehabilitation”, “cardiac physiotherapy”, “physiotherapy in frailty syndrome”, “rehabilitation in frailty”, “cardiac rehabilitation in frailty”, “physical activity in frailty syndrome”, “6MWT in frailty”, “rehabilitation among frail cardiac patients”, and “cardiac training”. Conditions of inclusion for the research corpus are: subjects are cardiac patients with accompanying frailty, an intervention based on CR, and assessment of the impact of CR or its modifications on frailty level. Exclusion conditions are: no frailty or pre-frailty syndrome, intervention without physical activity, assessment of the impact of CR does not concern the frailty level, the CR model is not applied or there are no references to that, and research published before 2014.

The retrieved articles were independently reviewed and were considered eligible if the two reviewers (MW and NSL) independently decided that they met the inclusion criteria described previously. Disagreements were resolved by consensus or by discussion and consultation of a third reviewer (KWT).

In the first stage, all records were identified from searches of the electronic databases. In the next stage, two researchers (MW and NSL) independently screened the titles and abstracts to identify potentially eligible studies and remove duplicates. In the third stage, studies that were potentially eligible were selected for full-text review. Disagreements were resolved by discussion to reach a consensus.

An initial database was developed, pilot-tested, and refined to ensure consistency with outcomes reported in the literature. Data were independently extracted from eligible articles by two reviewers. Data extraction discrepancies between the reviewers were resolved by consensus. The types of data recorded in the standardized data extraction forms included general manuscript information, study design, risk of bias using the Newcastle–Ottawa scale, patient characteristics, study characteristics, and intermediate- to long-term (≥6 months) main outcomes.

The main outcomes of interest for this review included the New York Heart Association (NYHA) functional class and frailty status measured by the FI and FP.

The present work follows the recommendations of the preferred reporting items for systematic reviews and meta-analyses (PRISMA) Statement [14]. In PROSPERO the review is registered as ID: CRD42024605406.

**Table 1:** Studies included to review.

Author, Study	Population	Rehabilitation Program	Effects
MacEachern, 2024 [15]	759 of 4000 adults in a stable clinical state (CAD, MI, HF, after PCI or CABG)	Conventional CR	↓FI—↑chance to achieve CR goals
Nagatomi, 2022 [16]	30 patients with HF (15 in intervention group and 15 control group)	30–40 min, 3–5/week aerobic training and 2–3/week resistance training with intensity on 11–13 on 20-point Borg scale; home-based rehabilitation with telemetry supervision	↑exercise tolerance ↑lower extremities strength
Reeves, 2016 [17]	360 patients ≥60 years hospitalized with ADHF	REHAB-HF project; hospital-stage rehabilitation and then 12 weeks of outpatient CR (60 min, 3/week) and then 4–6 months of individual walking and functional training	↑physical fitness ↓FP and FI ↓Geriatric Depression Scale ↓falls ↓readmissions ↑strength of lower limbs
Kitzman, 2021 [18]			
Pandey, 2023 [19]			

Mudge, 2021 [20]			
Nagai, 2018 [21]			
Tarazona-Santabalbina, 2016 [22]	51 frail elderly people	Training including strength, balance, flexibility and coordination exercises	↓FP ↑balance ↑physical fitness ↓need of medical advices
Beigienė, 2021 [23]	97 participants of CR; 3 groups; 2 intervention groups participating in individual resistance training	Traditional resistance training and training with mechanical devices	↑gait speed
Beigienė, 2021 [24]	63 participants of CR; 3 groups; 2 intervention groups participating in individual resistance training	Resistance training 3/week	↑functional capacity ↑physical performance Benefits comparable to endurance-based CR
Nakaya, 2021 [25]	226 elderly patients with ADHF hospitalized in Japan	Resistance and balance training	↑physical fitness ↑gait speed ↑endurance
Tamulevičiūtė-Prascienė, 2021 [26]	116 of 252 patients after valve surgery	3/week resistance training with free weights, resistance bands and workout machines additionally to standard CR	↑walk distance ↑gait speed ↑physical fitness ↑workload
Kato, 2021 [6]	28 elderly patients with HF	“Chair-stand” training with the HAL system; 6–10 days period, 5 to 30 min per day	↑walk distance ↑ physical fitness ↑muscle strength of lower limbs
Hall, 2023 [27]	Veterans with frailty qualified to abdominal and thoracic cardiac and non-cardiac surgeries	4–6 weeks before surgical intervention, veterans participated in tailored physical prehabilitation focused on strengthen skeletal muscles, inspiratory muscles training and endurance training.	↑gait speed ↓time of 5 chair raises ↓time of timed-up-and-go ↑Maximum and mean inspiratory and expiratory pressures
Sahar, 2024 [28]	74 patients awaiting CABG	8-week resistance training with cuff weights/dumbbells	↑physical effort ↑oxygen saturation heart rate normalization ↓clinical frailty score ↓NYHA

CAD—Coronary Artery Disease, MI—Myocardial Infraction, HF—Heart Failure, PCI—Percutaneous Coronary Interventions, CABG—Coronary Artery Bypass Grafting, CR—Cardiac Rehabilitation, FI—Frailty Index, ADHF—Acute Decompensated Heart Failure, FP—Frailty Phenotype, HAL—Hybrid Assistive Limb, NYHA—New York Heart Association functional class.

## 3. Results

Pursuant to the literature review, it can be noted that it is advantageous to apply stationary CR based on aerobic training supplemented with resistance and respiratory exercises and also supervised household rehabilitation in frail or pre-frail patients. One of the cardiovascular disease symptoms, besides anginal complaints, is a decline of physical effort tolerance measured by the 6 min walking test (6MWT) and VO2 peak (peak oxygen uptake). Results of these tests refer to the assessment of frailty (FI). The authors proved that these parameters are better indicators than muscle strength and can be independent predictors of frailty in the cardiac patient population [29,30].

### 3.1. Traditional Cardiac Rehabilitation

Patients with lower levels of frailty at admission to a rehabilitation program stand a better chance of improving their outcomes and achieving their individual goals of rehabilitation. A total of 4000 adults in a stable clinical state (coronary artery disease, myocardial infraction, or heart failure, after percutaneous coronary intervention or coronary artery bypass) who were eligible for post-hospital CR because of different cardiovascular diseases were examined by medical specialists over 10 years. They participated in educational meetings once a week and in physical training twice a week during a 12-week rehabilitation period. Training sessions lasted 60 min, including 40 min of aerobic training on a cycle or arm ergometer, 10 min of resistance exercises with dumbbells and resistance bands, and body weight exercises. Additionally, at home, they had to execute individual programs of physical activity with aerobic and resistance exercises prescribed by physiotherapists. The training intensity was set at 11–13 on a 20-point Borg scale. The authors of the research noted that rehabilitation and medical procedures focused on the reduction of frailty measured by the FI can contribute to more effective treatment of cardiovascular diseases [15].

Other research presents a home-based rehabilitation project for patients with heart failure and frailty syndrome with physical intolerance, one of the most burdened groups of patients, often not able to take part in outpatient rehabilitation. These were people with heart failure and accompanying frailty measured by a modified CHS scale who qualified for the research. Patients in the treatment group trained for 30–40 min each session, performing aerobic training 3–5 times a week and individual resistance training with body weight exercises 2–3 times a week. Furthermore, the average daily step number was counted. The intensity was set at 11–13 on the 20-point Borg scale. Nagatomi et al. [16] revealed that applying telemetry controlled home-based CR significantly improved the outcomes of the FI fitness tests, knee extension strength, and isometric tension in the group of patients with heart failure and frailty syndrome in comparison with the group performing traditional CR [16].

In light of home-based rehabilitation, the effectiveness of continuing the rehabilitation applied at the hospital-stage is worth mentioning. In the REHAB-HF project, between September 2014 and September 2019, there were 292 examined people, among whom 163 were classified as frail and 129 as pre-frail, according to Fried’s scale. Patients of the intervention group participated in the hospital-stage rehabilitation and, after discharge, they continued an outpatient CR for 12 weeks. They attended 60 min training sessions 3 times a week. The intensity was set at 10–15 RPE on the 6–20 Borg scale for endurance exercises and at 15–16 RPE for resistance exercises. Then, after 4 weeks of activity, the intensity target was set to ≥20 beats per minute above the resting heart rate. Each session included a warm-up, a main part with balance, mobility, strength, and endurance exercises, and a cool-down. After the second stage of rehabilitation, the patients of the treatment group had to continue walking and functional training on their own in 30–45 min sessions in the 4–6 months period. The control group patients did not participate in any post-hospital physical activity program [17]. After 3 months, outcomes showed an improvement in the frailty level and an improvement in physical fitness as measured by the SPPB (Short Physical Performance Battery), a 5 chair raises test, and walking speed among patients performing post-hospital rehabilitation versus those in the control group. After 6 months, the difference between groups was not substantial, but the authors observed an improvement in walking distance (6MWT), walking speed, and in the Geriatric Depression Scale and decline of the Fried frailty level in favor of the intervention group. In the final outcome, the rate of overall falls was lower (−33%) [18,19]. One of the components of the heart failure patient assessment is the frequency of readmission. It is noted in the research that a decline of the FP is associated with a decline in the risk of rehospitalization [19], which proves clinical stabilization and a slowing of disease progression. The improvement in the frailty scale attributed to home-performed resistance training was observed also by Mudge et al. [20], who carried out a program including elements of aerobic, resistance, and balance training among a group of 256 patients with heart failure (110 without frailty features and 146 with frailty syndrome). The program lasted 3 months with 2 training sessions per week. The intervention group patients performed physical exercises on their own at home while the control group executed center-based training. Both groups achieved similar results. What was observed was that people with higher frailty levels in the baseline 6MWT covered shorter distances than the less frail patients, but, after 6 months, they achieved better outcomes than non-frail participants. Moreover, persons who were basically frailer improved their FI scale outcome independently of the form of implemented rehabilitation. In the intervention group as well as the control group, patients with initially higher levels of frailty achieved the program goals and a reduction of frailty more often than the non-frail participants [20]. Nagai et al. [21] indicated their reasoning for the increase of daily physical activity in frail patients. The authors compared the group of patients performing resistance training (control group) to patients who, additionally, were increasing their number of steps on a daily basis and were limiting sedentary time (the intervention group). In the treatment group, an increase in the number of steps per day, the improvement of strength of lower limbs, and a decrease in frailty scores were achieved. Although the general frailty status and the quality of life assessment did not change substantially, the authors suggest that increasing the level of daily physical activity helps to reduce frailty level and to improve the mobility of frail people [21].

### 3.2. Multicomponent Training

Multicomponent training used in frail patient treatment shows positive effects. Tarazona-Santabalbina et al. [22] subjected 51 frail elderly people, i.a., the ones with cardiovascular diseases, to training that included strength, balance, flexibility, and coordination exercises. In 31.4% of these patients, the frailty measured by the FP was reversed, in contrast to patients in the control group in which these changes were not observed. The improvement of physical fitness included balance and stability measured on the Barthel and Lawton scales, and the training results were evident in the Tinetti test, because being an independent and self-reliant person is an important aspect of quality of life for elderly people. The significant effects of the program were observed also in the Physical Performance Test (PPT). Amongst people performing training, the need for medical advice decreased. Furthermore, in final assessment (after 6 months), those patients who did not execute multicomponent training achieved inferior results in the functional test than in the initial examination [22]. Complementing CR based on endurance training with resistance and balance exercises in patients with frailty and pre-frailty syndrome has supplementary effects, like an improvement in gait speed, regardless of what equipment was used for training. This was proved by Beigiene et al. [23,24], who examined 97 people divided into three groups: a control group, with typical CR, and two intervention groups—one using the equipment and the other using devices like BIODEX and HUR. Patients of the all three groups increased walking speed, but this change—from normal gait (<1 m/s) to a fast walk (≥1 m/s)—was more effective during short-term CR in the group with modified training [23,24]. The need for multicomponent rehabilitation for patients with acute decompensated heart failure (ADHF) (heart failure with a reduced ejection fraction and also heart failure with a preserved ejection fraction) was also proven by Nakaya et al. [25], who compared the group realizing standard CR focused on locomotion with the group performing additional resistance and balance exercises and cycle ergometer training as well. Patients of intervention group improved their physical fitness, gait speed, and endurance. In contrast, the parameters in the control group did not change or get worse. The authors indicate that multicomponent physical activity effectively enables improvement of the fitness of elderly people with ADHF regardless of the its type. Indeed, abandonment of this training leads to functional decline [25].

Tamulevičiûte-Prasciene et al. [26] describe research carried out between January 2018 and November 2019 in which 252 patients took part after valve surgery. Of these, 116 met the requirements for inclusion. The control group was composed of 56 persons who participated in the CR, a combination of cycle ergometer training, aerobic exercises, and breathing muscle exercises. The intervention group included 60 patients who, 3 times a week, frequented sessions of resistance training with the use of free weights, resistance bands, and workout machines in addition to standard CR. In the short-term assessment, all measured parameters (6MWT, gait speed, SPPB, and workload) except for peak VO2 were improved. The changes of all parameters were significant after 3 months of training. Both short-term and medium-term results between groups were not significant. However, the assessment of frailty status was meaningfully improved in the intervention group compared to the control group [26].

### 3.3. Using a HAL (Hybrid Assistive Limb) Exoskeleton in CR

Using new technologies in the physical activity-based therapy of patients with heart failure can be beneficial given the presence of myopathies. Kato et al. [6] subjected two groups of patients to “chair-stand” training. The exercise was performed in a 6–10 day period, for 5 to 30 min per day (with a gradual increase in training time each day). One group of patients practiced fully on their own, the other with the help of a HAL. Participants of both groups extended their walk distance by about 60 m in the 6MWT attempt and improved their physical fitness as measured by the SPPB. The authors underline that applying a HAL system in the “chair-stand” exercise can effectively strengthen the muscles of the lower extremities in cardiac patients with frailty syndrome which manifests itself, i.a., as a decrease in muscle strength and mass, and is a predictor of heart failure [6].

### 3.4. Prehabilitation

Prehabilitation is a form of therapy which aims to optimize or prevent the decline of physical fitness as part of the preparation for other medical interventions. Minimizing the risk of post-interventional complications and accelerating health restoration are others of its goals [31]. The beneficial impact of prehabilitation is observed in cardiac patients with associated frailty syndrome.

Veterans with at least mild frailty (RAI—Risk Analysis Index) who qualified for coronary artery bypass grafting (CABG), valve surgeries, non-cardiac thoracic surgeries, or abdominal surgery were tested by Hall et al. [27]. In the 4–6-week period before surgical intervention, these veterans participated in tailored physical prehabilitation and nutrition education meetings; they were also given supplementation. Exercises focused on increasing skeletal muscle strength (resistance bands and body weight exercises), inspiratory muscles training (IMT), and coordination (balance exercises and reeducation of body position changes), and endurance training (walking or cycle ergometer). Early sessions were realized in the hospital under telemetric control. Then, patients performed their training twice a week in outpatient conditions or at home, supervised by phone. They had to perform 60 min of training daily, strength training three times a week, and other forms of activity five times a week. They were tested every week until surgery, and 30 and 90 days post discharge. On the day of surgery, an improvement in physical fitness compared to the day of program eligibility was observed. However, after 90 days, the fitness level was reduced to the initial outcomes; the exception was gait speed, which was increased in the final test [29]. This shows how important it is for elderly patients to continue physical activity on their own in the post-operative period to maintain the effects of prehabilitation. In another project, researchers proposed 8 weeks of resistance training with cuff weights/dumbbells or conventional CR to patients with frailty. After surgery, the measured parameters were improved in both groups, but the change noted in the resistance training group was greater. Patients of this group achieved better tolerance of physical effort assessed in 6MWT, oxygen saturation and heart rate normalization. Furthermore, the symptoms of heart failure were relieved in the NYHA class, and the clinical frailty score and essential frailty toolset were improved in tests just before CABG and three days after it [28].

## 4. Discussion

When searching the PubMed database to find publications concerning the impact of directed physical rehabilitation on frailty level in cardiac patients, it turned out there is not much research on this subject. Most of the studies concern multicomponent training, and prove its beneficial effects. This fits into assumptions of comprehensive cardiac rehabilitation, which includes not only endurance aerobic training, but also elements of resistance and respiratory training and self-directed physical activity. Part of the articles regard rehabilitation continued at home. These papers prove that it is important to teach patients about the impact of physical activity on their health, and that it is crucial to continue rehabilitation after hospital discharge because cessation contributes to a reduction in the achieved improvement, to a decline in fitness, and to an increase in physical intolerance.

Frailty is a changeable agent of chronic disease [7,19,22]. The British Geriatric Society recommends, i.a., physical activity (particularly resistance exercises as a complement to aerobic and general fitness training) for the prevention and restoration of physical fitness, as a lack of it is an essential component of frailty syndrome. It is also recommended to implement diet and pharmacotherapy and to quit smoking and drinking alcohol [32]. INSERM (Institut Nationale de la Santé et de la Recherche Médicale) experts recommend educating elderly people about frailty and the implementation of rehabilitation and adapted physical activity. They aims to reduce the risk of the appearance the frailty syndrome or the limitation of its effects (like a decline in functional fitness and a resulting decrease in quality of life, and a minimizing of the frequency of falls and their sequalae) [33].

In other metanalyses, it has been proved that CR positively acts on the fitness of frail patients and on betterment of the frailty score. It has also been remarked that participating in rehabilitation reduces the risk of rehospitalizations because of heart failure causes, and people with poor frailty at rehabilitation admission have a lower chance of completing the CR program. Frail people with cardiovascular diseases have twice the morbidity and mortality rate than those without frailty syndrome. Initially high frailty level is connected to poor health and greater disease progression. However, frailty is a modifiable factor. The implementation of CR has a favorable impact on the improvement of the frailty score and physical health in patients with mild to severe frailty [34,35]. Resistance training helps to increase muscle strength and exercise capacity, and, when appropriately couched and supervised, is safe and can be an effective component of CR by mitigating risk factors for cardiovascular events and reducing disability in seniors [35]. Multicomponent training, including, i.a., resistance exercises, is recommended for frail people, because it improves cardiorespiratory fitness, balance, and strength, which results in minimizing fall risk and the associated risks, and also improves functionality [36]. A comprehensive program of physical activity composed of gradual implementation of resistance, balance, mobility, and endurance training is the most effective instrument to fight frailty syndrome in cardiac diseases. Even if physical activity is not able to stop the ageing process, it is the most effective method of prevention and reduction of the level and of slowing-down the progress of frailty syndrome, especially in patients with cardiac diseases. Moreover, in the opinion of Vazquez-Guajardo et al. [37], physical rehabilitation should be prescribed like medication—including specificity, frequency, intensity, volume, and progression [37,38]. In another review, similar conclusions are presented; the authors remark that frailty is not a contraindication to physical activity, rather it is the most serious reason for it [39]. Afilalo notices that early commencement of rehabilitation after an acute cardiac event interrupts the process of deconditioning [39].

It is worth remarking that CR influences many systems of the human body, allowing for, i.a., a greater general physical tolerance, an increase in muscle mass, and an ameliorated cardiorespiratory system, which can lower the frailty level. Participating in CR results in a reduction of cardiac-caused mortality and of a recurrence of myocardial infraction and restenosis as well as a reduction of the frequency of repeat CABG and PCI [40]. Among patients with atrial fibrillation, the decline in mortality and rehospitalizations was connected to improvements in aerobic capacity. It was observed that CR contributes to the improvement of cardiometabolic health and aerobic and functional capacity [41]. The authors of another review inferred that CR is advantageous for patients after valve surgeries, particularly for short-term physical capacity [42].

Frailty syndrome is common in the heart failure population and is associated with poor disease prognosis. Testing the patients with 6MWT or Fried’s scale before hospital discharge helps to identify those at greater risk of complications and readmission [43]. Indeed, integration of the concept of frailty in CR allows for the identification of those patients who need a customized approach. It helps not only to improve cardiopulmonary fitness, but also to decrease sarcopenia and overall frailty. One common program dedicated to all elderly patients is not adequate for such a heterogeneous group as the elderly [39], who are often burdened with cardiovascular diseases.

### Practical Implications

With the growing elderly population, it is worth ensuring that as much of this population as possible is made up of independent people who do not require the care of others. This will help optimize health policy and not cause additional financial expenses for patients’ families and the state. Reducing the frailty status in cardiac patients by implementing prehabilitation diminishes surgical risks and the time needed to recover from surgery. There is a lower risk of readmissions in patients without or with reduced frailty. Greater physical fitness is associated with lower fall risk and related complications.

## 5. Summary

Comprehensive rehabilitation is essential for a betterment of the clinical state of frail patients. Complex CR should be composed of resistance, mobility, balance, and respiratory training as well as endurance training. Increasing the daily physical activity level and encouraging elderly people to perform training programs at home is crucial for their fitness. It is necessary for them to do resistance exercises, which make muscles stronger, which transfers into walking safety in the frail elderly. CR can be used to modify the changeable factors of developing frailty syndrome.

### Study Limitations

Despite the relatively large number of patients assessed, the most important limitation of our systematic review was the small number of studies included. This is partly due to the fact that some original papers did not meet the inclusion criteria. Another limitation of the study is associated with the various methods used by authors to assess physical fitness and frailty status, and the heterogeneity of the study group, which makes it very difficult to compare outcomes and state the greatest improvement in one study versus another study. Moreover, the included studies did not take into account the patient point of view about rehabilitation, and attention was directed only to the impact of rehabilitation on frailty, excluding other outcomes.

## Data Availability

The data are contained within the article.
